# Discovery and Characterization of a Thermostable and Highly Halotolerant GH5 Cellulase from an Icelandic Hot Spring Isolate

**DOI:** 10.1371/journal.pone.0146454

**Published:** 2016-01-07

**Authors:** Dimitra Zarafeta, Dimitrios Kissas, Christopher Sayer, Sóley R. Gudbergsdottir, Efthymios Ladoukakis, Michail N. Isupov, Aristotelis Chatziioannou, Xu Peng, Jennifer A. Littlechild, Georgios Skretas, Fragiskos N. Kolisis

**Affiliations:** 1 Institute of Biology, Medicinal Chemistry & Biotechnology, National Hellenic Research Foundation, Athens, Greece; 2 Laboratory of Biotechnology, School of Chemical Engineering, National Technical University of Athens, Athens, Greece; 3 Henry Wellcome Building for Biocatalysis, Biosciences, College of Life and Environmental Sciences, University of Exeter, Exeter, United Kingdom; 4 Danish Archaea Centre, Department of Biology, Copenhagen University, Copenhagen, Denmark; Consejo Superior de Investigaciones Cientificas, SPAIN

## Abstract

With the ultimate goal of identifying robust cellulases for industrial biocatalytic conversions, we have isolated and characterized a new thermostable and very halotolerant GH5 cellulase. This new enzyme, termed CelDZ1, was identified by bioinformatic analysis from the genome of a polysaccharide-enrichment culture isolate, initiated from material collected from an Icelandic hot spring. Biochemical characterization of CelDZ1 revealed that it is a glycoside hydrolase with optimal activity at 70°C and pH 5.0 that exhibits good thermostability, high halotolerance at near-saturating salt concentrations, and resistance towards metal ions and other denaturing agents. X-ray crystallography of the new enzyme showed that CelDZ1 is the first reported cellulase structure that lacks the defined sugar-binding 2 subsite and revealed structural features which provide potential explanations of its biochemical characteristics.

## Introduction

Cellulose is the most abundant biopolymer on Earth, with about 100–1000 trillion tons being naturally produced in the form of plant biomass every year [1, 2]. It is considered to be an almost inexhaustible source of raw material, which can be transformed through biotechnology-based manipulations to environmentally friendly products of high value, such as papers, textiles, animal feed stocks, biofuels and others [3]. On one hand, cellulose is a polymer of simple composition, comprised of D-glucose units connected with β-1,4 glycosidic bonds [4]. On the other hand, tight packing of these linear chains and the formation of a rigid crystalline structure make cellulose an extremely difficult starting material, which is resistant to decomposition into smaller, more manageable units which can be further transformed into useful products. In nature, cellulose is degraded enzymically by the concerted activity of three different types of glycosyl hydrolases: (i) endo-1,4-β-glucanases (cellulases) cleave the internal bonds of the cellulose polymer randomly, (ii) exo-1,4-β-glucanases, attack the reducing or non-reducing end of the cellulose chain, and (iii) β-glucosidases convert cellobiose, the main product of the endo- and exo-glucanase activity, to glucose [5].

In industrial applications, cellulosic starting materials can be depolymerized either by chemical or enzymic means, or by a combination of both [6]. Because of the ability of cellulose–degrading enzymes to “access” the recalcitrant structure of cellulose in a low-energy and environmentally friendly manner, purely chemical processing of lignocellulosic biomass is being replaced by enzymic methods wherever possible. Owing to their central role in these processes, the industrial application of cellulases is of great value and the US Department of Energy has projected that cellulases will become industrial blockbusters, reaching an annual market share of about $ 9 billion by the year 2030 [7]. One of the most important factors limiting the wide industrial use of cellulases is the fact that these enzymes need to perform under harsh conditions, such as high temperature, high salinity, presence of organic solvents and detergents which can all cause protein denaturation. Under such conditions, the vast majority of the available enzymes perform very poorly. Therefore, new and improved enzymes with the ability to retain their catalytic activity in such “industrial environments” need to be identified.

Two strategies can be employed to obtain better biocatalysts. The first is protein engineering, either through rational design or directed evolution [8–10], an approach which has presented numerous successes [11–13]. The second strategy is mining nature’s genetic reservoir, whereby genes that encode enzymes with novel properties can be identified from the DNA extracted from previously uncharacterized organisms either bioinformatically or by functional screening [14]. Again, several examples of this approach which has led to the discovery of novel enzymes have been reported [15–18]. Extremophilic organisms are a very rich source for such enzymes, as they have evolved to thrive in extreme environments. Culturing or culture–independent approaches are applied to retrieve genomic or metagenomic material from extreme habitats. DNA isolation can then be followed by functional or bioinformatics screening which can reveal novel enzymes with the desired properties [19, 20].

In this study, as part of the EU 7^th^ Framework Program project “Hotzyme” (http://hotzyme.com/), we aimed to identify novel thermostable polysaccharide-degrading enzymes with properties suited for industrial applications. Initially, we carried out an enrichment approach to access microorganisms which can degrade polysaccharides using a sample collected from a hot spring located in Iceland. Then, DNA isolated from this source was sequenced on a next-generation sequencing platform and subjected to bioinformatic analysis to identify sequences encoding for putative cellulolytic enzymes. By following this approach, we identified a new thermostable and extremely halotolerant GH5 cellulase, termed CelDZ1. This novel enzyme was cloned and overexpressed in *Escherichia coli* and has been thoroughly characterized both biochemically and structurally. CelDZ1 exhibits a catalytic profile that renders it a potentially attractive industrial biocatalyst. From a structural point of view, CelDZ1 is quite unique among its analogues in that it lacks the sugar-binding 2 subsite which is conserved in all known related enzymes.

## Results and Discussion

### Enrichment culture and taxonomic analysis

The outflow of a hot spring in Grensdalur, Iceland (64°01'53.4"N, 21°11'50.4"W) was sampled, enriched anaerobically with 0.5% xylan at 55°C and pH 7.0 and serially diluted to get a pure isolate. The 16S rRNA fragment was amplified from the extracted genomic DNA and sequenced. The sequence of the gene fragment was then searched against the NCBI database and showed 99% identity to *Thermoanaerobacterium*. The sequencing reads were also assigned to taxa using the MEtaGenome ANalyzer (MEGAN) [21], which assigned the reads to either *Thermoanaerobacterium thermosaccharolyticum* or *Thermoanaerobacterium xylanolyticum*, thereby verifying that the gene originates from a *Thermoanaerobacterium* species.

### Discovery of CelDZ1

**Fig 1:**
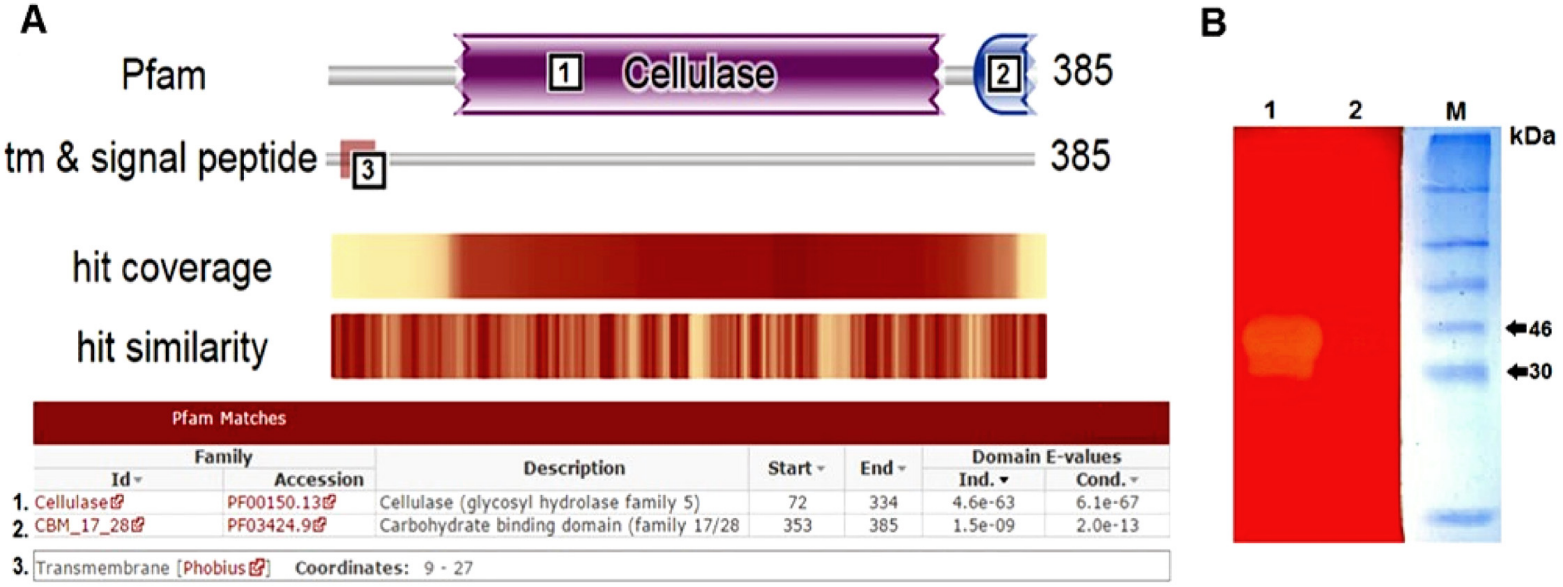
Discovery of the cellulolytic enzyme CelDZ1. **(A)** The amino acid sequence of the putative cellulolytic enzyme corresponding to the *celDZ1α* ORF was analysed against the Pfam-A database. The analysis revealead that the predicted sequence consists of a GH5 catalytic domain (denoted as domain 1), a carbohydrate-binding module (CBM) 17/28 (denoted as domain 2) and a transmembrane anchor (denoted as domain 3). **(B)** Zymogram analysis for the detection of cellulolytic activity via SDS-PAGE analysis and Congo red staining of a CMC-containing acrylamide gel. M: molecular weight marker; 1: cell lysate producing the target protein 2: cell lysate carrying an empty vector.

Genomic DNA isolated from the xylan-degrading culture described above was sequenced using an Illumina next-generation sequencing platform and the data were uploaded for the subsequent bioinformatic analysis to our customized metagenomic data analysis platform termed ANASTASIA (Automated Nucleotide Aminoacid Sequences Translational plAtform for Systemic Interpretation and Analysis) (manuscript in preparation). Reads were assembled into larger sequence constructs (contigs) and examined for the presence of open reading frames (ORFs) possibly encoding for polysaccharide-degrading enzymes. From this analysis, a specific sequence that consisted of 385 amino acid residues, had a predicted molecular mass of 43.2 kDa and presented 59% identity to a previously characterized endoglucanase from *Bacillus akibai* [22] was selected for further investigation. Sequence analysis against the Pfam-A database [23] using HMMER [24] revealed that the sequence contains two distinct putative functional domains: a glycosyl hydrolase family 5 (GH5) catalytic domain according to the Carbohydrate-Active enZYmes database (CAZy) classification system [25], and a 17/28 carbohydrate-binding module (CBM) (Fig 1A). Analysis of the amino acid sequence on the TMHMM Server [26] predicted the existence of a putative transmembrane helix at the N terminus of the protein (amino acids 9–27), and its catalytic domain (amino acids 28–385) to be facing outward from the membrane (Fig 1A).

The identified ORF, designated as *celDZ1α*, was amplified (along with a C-terminal hexahistidine tag) by PCR from genomic DNA isolated from the aforementioned xylan-degrading culture and was cloned into the plasmid pET-28a(+) to form the vector pET-CelDZ1α. *E*. *coli* BL21(DE3) cells were transformed with pET-CelDZ1α, grown in LB medium at 37°C with shaking until the culture reached an optical density at 600 nm of 0.5, at which point 0.2 mM isopropyl thio-β-D-galactoside (IPTG) was added to induce *celDZ1α* overexpression. After additional incubation at 37°C for 4 hours, the cells were lysed by brief sonication and the proteins contained in 10 μl of the total cell lysates were separated (without prior boiling) by sodium dodecyl sulfate polyacrylamide gel electrophoresis (SDS-PAGE) on a gel containing 0.25% carboxymethyl cellulose (CMC) as a potential substrate for degradation. After staining of the gel with Congo red and destaining, a zone of discoloration appeared at an apparent molecular mass of about 42 kDa (Fig 1B), indicating that *celDZ1α* encodes a protein with cellulolytic activity. In order to verify that the observed discoloration occurred due to the ability of the enzyme to degrade CMC, we used an alternative method to detect cellulolytic activity, which is based on the colorimetric detection of released reducing sugars from CMC using 3,5-dinitro-salicylic acid [27]. Lysates of *E*. *coli* cells overexpressing CelDZ1α from pET-CelDZ1α yielded a rapid colour change from yellow to orange, while lysates from cells carrying the empty vector did not, thus supporting the CMC-degrading ability of CelDZ1α (data not shown).

### Purification and biochemical characterization

As mentioned above, CelDZ1α is predicted to be a membrane-bound enzyme containing an N-terminal, single-pass trans-membrane helix. In order to study the biochemical properties of the new enzyme, we cloned a modified *celDZ1α* gene encoding a truncated version of the resulting protein, which is expected to be produced in soluble form. In this truncated construct, henceforth referred to as *celDZ1*, the sequence encoding for the first 27 amino acids of CelDZ1α was replaced with a hexa-histidine tag and the gene was inserted again into pET-28a(+) to form plasmid pET-CelDZ1. Expression tests with the two constructs showed that CelDZ1 accumulated at higher levels, and was more soluble and less prone to degradation compared to the original full-length protein (data not shown). For these reasons, the construct pET-CelDZ1 was chosen for all subsequent biochemical studies. Overexpression of CelDZ1 in *E*. *coli* BL21(DE3) cells resulted in the accumulation of primarily soluble enzyme, which could be purified via immobilized metal affinity chromatography to near homogeneity (data not shown).

CelDZ1 was found to be highly active against soluble polymeric substrates containing β-1,4 glycosidic bonds, such as CMC (74 U/mg) and β-D-glucan from barley (589 U/mg) (Table 1). On the contrary, no activity could be detected with insoluble cellulosic substrates, such as Avicel and filter paper. Furthermore, CelDZ1 did not exhibit β-glucosidase activity as it was inactive against cellobiose. Also, the enzyme was unable to hydrolyze the β-1,3-linked substrate laminarin and displayed no activity against xylan, galactomannan and pectin (Table 1). Thus, we conclude that CelDZ1 is a novel endo-glucanase for soluble cellulose.

**Table 1:** Substrate specificity of CelDZ1.

Substrate	Main linkage type	Solubility	Specific activity (U/mg)
CMC	(β-1,4) Glc	soluble	74 ± 9
β-D-glucan	(β-1,3/4) Glc	soluble	589 ± 7
Cellobiose	(β-1,4) Glc	soluble	undetectable
Laminarin	(β-1,3) Glc	soluble	undetectable
Galactomannan	(β-1,4) Man	soluble	undetectable
Pectin	(α-1,4) Gal	soluble	undetectable
Xylan	(β-1,4)Xyl	soluble	undetectable
Avicel	(β-1,4) Glc	insoluble	undetectable
Filter paper	(β-1,4) Glc	insoluble	undetectable

**Fig 2:**
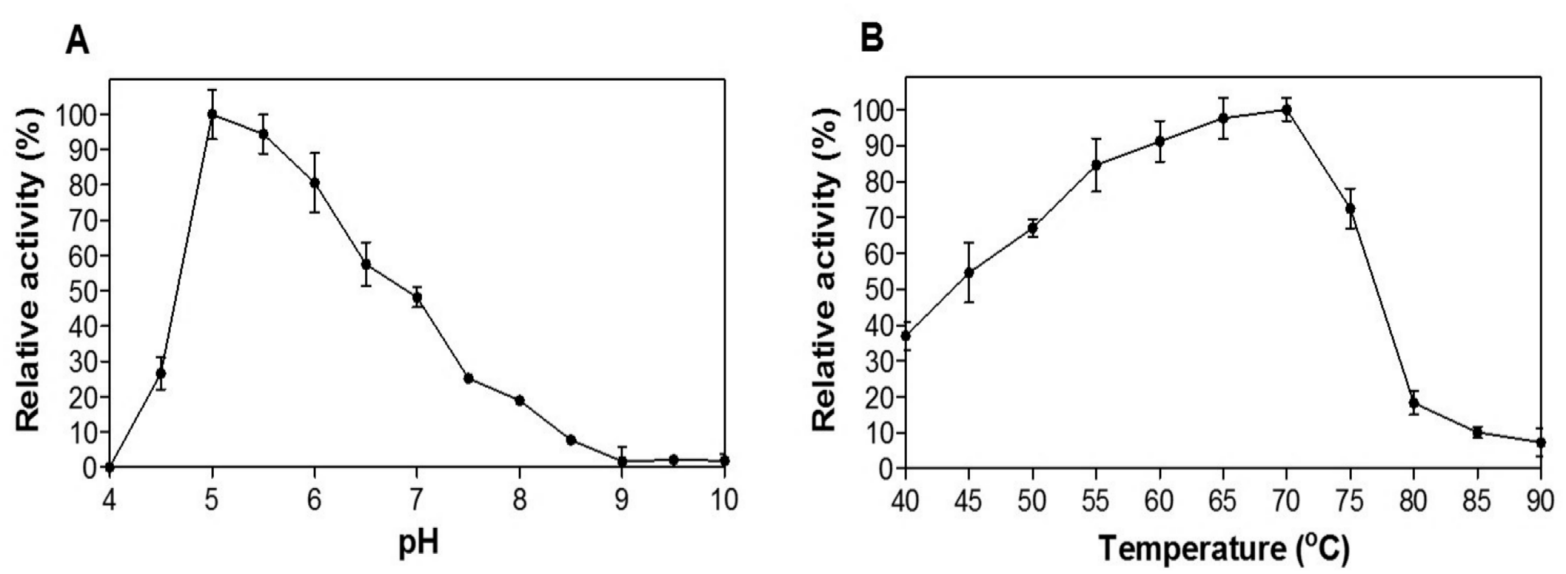
Effect of pH and temperature on the activity of CelDZ1. **(A)** CelDZ1 activity was measured in the standard reaction at 40°C for 5 min at pH values ranging from 4 to 10 and **(B)** at temperatures between 40 and 90°C for 5 min in a pH 5 buffer. The reported values correspond to the mean value from three independent experiments performed in triplicate and the error bars to one standard deviation from the mean value.

Further biochemical characterization of CelDZ1 was carried out using CMC as a substrate. First, we determined the optimal pH and temperature for CelDZ1 cellulolytic activity. The enzyme was assayed within the pH range of 4–10 at 40°C and pH 5 was found to be the optimal value for CelDZ1 activity (Fig 2A). At pH values 6 and 7 the relative activity of the enzyme was 80% and 48% of its maximal level, respectively, while below pH 4 and above pH 9 CelDZ1 was inactive. This indicates that CelDZ1 is an acidophilic cellulase, similarly to what has been reported for the homologous cellulase Cel5A from *Thermoanaerobacter tengcongensis* MB4 [28]. Interestingly, however, this is in contrast to CelDZ1’s closest sequence homologue from *B*. *akibai* [22] and closest structural homologue CelK from *Bacillus* sp. KSM-635 (see below) [29], which are both alkalophilic with pH optima of 9 and 9.5, respectively.

CelDZ1 has a broad temperature range of action as it retains significant levels of cellulolytic activity at temperatures between 40 and 80°C, with its optimal temperature found to be 70°C (Fig 2B). At its optimal conditions, CelDZ1 hydrolyzed CMC following Michaelis-Menten kinetics with a K_M_ and k_cat_ value of 6.1 ± 0.9 mg.ml^-1^ and 46.3 s^-1^, respectively. K_M_ is expressed here in terms of mass instead of moles due to the natural heterogeneity of the substrate. Based on these values, k_cat_/K_M_ was determined to be 7.6 mg^−1^.ml.s^−1^, a catalytic efficiency value which is very close to those reported for other related cellulases, such as CelE1 [15], Cel5A and its engineered variants [12].

**Fig 3:**
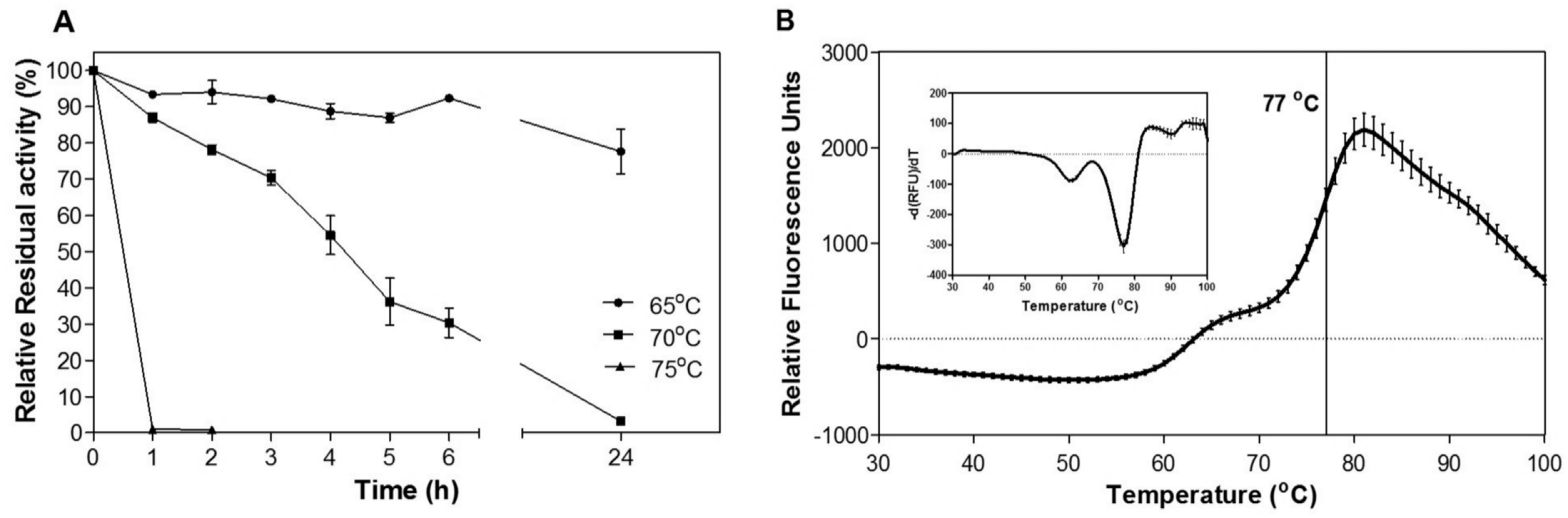
Thermostability of CelDZ1. **(A).** Catalytic thermostability of CelDZ1 evaluated by measurements of residual CMC-degrading activity after high-temperature exposure at 65, 70 and 75°C for up to 24 h. **(B).** Thermal denaturation analysis of CelDZ1 using differential scanning fluorimetry with the conformation-sensitive dye SYPRO Orange. The reported values correspond to the mean value from three independent experiments performed in triplicate and the error bars to one standard deviation from the mean value.

CelDZ1 was found to have very good stability when exposed to high temperatures, as determined by measurements of residual levels of cellulolytic activity after the enzyme was submitted to prolonged high-temperature incubations. Only a small change in catalytic efficiency could be detected after 24 h exposure at 65°C, whereas the enzyme retained more than 50% of its activity for at least 4 hours at 70°C (Fig 3A). At temperatures above 75°C the enzyme was rapidly inactivated. The thermostability of the enzyme is an important issue for its putative use in second-step processing of biomass at high temperatures. Thermal denaturation experiments using differential scanning fluorimetry (DSF) indicated a melting temperature (T_m_) of about 77°C (Fig 3B), which is consistent with the reported thermostability measurements for catalytic activity. Since thermal denaturation of CelDZ1 at temperatures higher than 65°C appears to be irreversible (Fig 3A), this T_m_ corresponds to the apparent midpoint melting temperature. A pre-transition state is also observed at 62°C, which could be attributed to partial unfolding of CelDZ1 due to thermal denaturation of the CBM that may be undergoing thermal unfolding independently of the catalytic domain.

**Fig 4:**
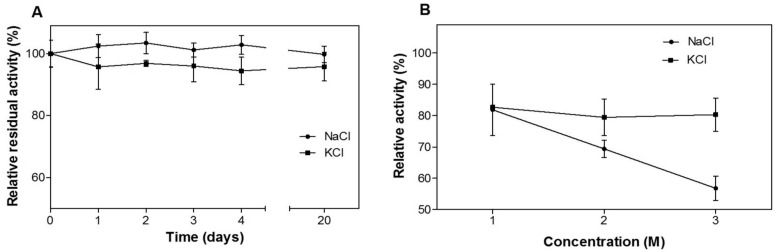
Halostability and halotolerance of CelDZ1. **(A)** CelDZ1 was incubated in 5 M NaCl and 4 M KCl for up to 20 days. At different time intervals aliquots were taken and the residual activity of the enzyme was measured in the standard reaction. **(B)** The activity of CelDZ1 in the presence of different high-salt concentrations was measured in the standard reaction. The reported values correspond to the mean value from three independent experiments performed in triplicate and the error bars to one standard deviation from the mean value.

Interestingly, CelDZ1 was found to be extremely stable in the presence of high salt. The enzyme’s catalytic activity remained practically intact after incubation for several days at near-saturating concentrations of NaCl and KCl (Fig 4A), while it also exhibited high levels of cellulolytic activity in aqueous solutions containing up to 3 M NaCl and KCl (Fig 4B). Interestingly, the enzyme retains about 80% of its maximal activity at KCl concentrations between 1 and 3 M but a monotonic decrease in activity (from 80% at 1 M to about 60% at 3 M) is detected in the presence of NaCl at the same concentration, thereby demonstrating the differential effect of the two cations on the thermo/halostability. Halostability and halotolerance are important properties for industrial enzymes, especially for those participating in the processing of biomass where the extraction of cellulose from lignocellulosic materials involves strong alkali pretreatment followed by neutralization with acid solution which result in the formation of high amounts of salts [30]. Even though a number of thermostable cellulases are halostable and overall polyextremophilic, few are truly halotolerant and can perform catalytic transformations efficiently in high-salinity environments [31]. Quite surprisingly, CelDZ1 is such an example of a highly halotolerant cellulase, despite the fact that it was not isolated from an organism derived from a saline environment.

Finally, we tested the effects of a range of metal ions, reducing agents, detergents and organic solvents on the cellulolytic efficiency of CelDZ1. When LiCl_2_, CaCl_2_, CuCl_2_ and ZnCl_2_ were added at 1 mM, CelDZ1 activity was not affected, whereas the addition of FeCl_2_ resulted in a minor reduction of cellulolytic activity (Table 2). Interestingly, the presence of MnCl_2_ stimulated CMC hydrolysis, thus suggesting that CelDZ1 may be a metalloenzyme. However, no metal ion bound to the enzyme was found in the solved crystal structure (see below) and EDTA did not have an inhibitory effect on its enzymic activity (Table 2). The presence of non-anionic surfactants such as Tween 20 and Tween 40 did not impact cellulolytic activity significantly when added at 1%, whereas Triton X-100 caused a significant loss in catalytic activity. Furthermore, after the addition of the anionic detergent SDS at the same concentration, the enzyme retained about 20% of its activity (Table 2). Interestingly, the addition of β-mercaptoethanol (βME) doubled the catalytic efficiency of the enzyme. Such an effect has been reported previously for other polysaccharide-degrading enzymes and has been attributed to the reducing effect of βME on the disulfide bonds between cysteine residues [32–34]. CelDZ1, however, does not contain cysteines in its amino acid sequence but the stimulation of its activity by βME could be a result of its protective effect against oxidation of the methionine residues present in CelDZ1 [35]. Lastly, CelDZ1 was found to be tolerant to the presence of organic solvents: in the presence of 1% methanol and ethanol CelDZ1 activity was practically unaffected, while the enzyme retained significant levels of activity when these alcohols were added at 5%. In aqueous solutions containing 10% methanol or ethanol, CelDZ1 retained only marginal levels of cellulolytic activity (Table 2).

**Table 2:** Effect or metal ions and other denaturing agents on the activity of CelDZ1.

Metal ion or chemical agent	Concentration	Relative activity (%)
None	-	100 ± 1
K+	1 mM	98± 1
Mn^2+^	1 mM	175 ± 1
Ca^2+^	1 mM	107 ± 2
Zn^2+^	1 mM	91 ± 6
Li^2+^	1 mM	97 ± 1
Mg^2+^	1 mM	102 ± 2
Na^+^	1 mM	95 ± 4
Fe^3+^	1 mM	27 ± 1
Cu^2+^	1 mM	75 ± 1
EDTA	1%	105 ± 6
β-Mercaptoethanol	1%	229 ± 17
Triton-X100	1%	64 ± 5
Tween 20	1%	102 ± 8
Tween 40	1%	102 ± 12
SDS	1%	21 ± 1
Methanol	1%	94 ± 1
Methanol	5%	68 ± 6
Methanol	10%	23 ± 2
Ethanol	1%	89 ± 4
Ethanol	5%	57 ± 5
Ethanol	10%	15 ± 1

### Structural analysis

#### Quality of the model

The CelDZ1α structure has been determined and refined to an *R*-cryst/*R*-free of 19.3/23.4% for all data to 1.9 Å without σ cutoff (Table 3). The N-terminal 49 amino acids forming the transmembrane helix and a linker to the catalytic domain were not defined due to disorder in the crystal. Out of the four monomers that make up the asymmetric unit of the enzymes crystal only two monomers, A and B, were clearly defined in the electron density since they are more restricted by many crystal contacts. The other two monomers, C and D, form fewer crystal contacts and are less well ordered. The loop formed by amino acid residues 120–125 in subunit D could not be modelled. Several C-terminal residues were also not modelled in all four of the monomers (Table 3).

**Table 3:** The data processing and refinement statistics for CelDZ1.

Crystal	Native
Beamline (Diamond)	I04-1
Resolution (Å)	36.38–1.88 (1.93–1.88)^a^
Wavelength (Å)	0.9174
Space group	C2
Cell dimensions	a, b, c = 107.3, 137.5, 121.3 Å; α, γ = 90° β = 114.4°
No. of protomers in A.U.	4
Solvent content (%); V_M_ (Å^3^ Da^-1^)	47; 2.36
Unique reflections	126320
Redundancy	3.5 (3.2)
Completeness	97.5 (90.0)
<(I)/ σ (I)>	17.0 (1.8)
R_sym_ (%)	3.6 (64.1)
Overall R-factor (%)	19.3
R_free_ (5% total data) %	23.4
Residues modelled	A (50–380); B (50–381);C (50–380); D (50–119; 126–383)
No. of waters modelled	585
RMSD bond length (Å)	0.012 [0.019] ^b^
RMSD bond angle (°)	1.48 [1.96]
Wilson B factor (Å^2^)	41.4
Average B factor	
Protein (Å^2^)	42.6
Water (Å^2^)	47.6
REFMAC RMS error estimate (Å)	0.148
Ramachandran analysis (% of residues)	0
Most favoured	86.9
Additionally allowed	13.1
Generously allowed	0.0
Disallowed	0
G-factor	0.0

^a^ Values for the outer resolution shell are given in parentheses.

^b^ Target values are given in brackets. R_sym_ = ∑_h_∑_J_|<I_h_>-I _J_(h) |/∑_h_∑_J_I(h), where I(h) is the intensity of the reflections h, ∑_h_ is the sum over all the reflections and ∑_J_ is the sum over J measurements of the reflections. R_cryst_ = ∑||Fo|-|Fc||/∑|Fo|. Wilson B-factor was estimated by SFCHECK [37]. The Ramachandran plot analysis and G-factor calculation were performed by PROCHECK [36].

The CelDZ1 model also contained 585 ordered water molecules and several ordered isopropanol, ethylene glycol and polyethylene glycol molecules that were present in the crystal cryoprotectant. The number of ordered solvent molecules was limited in comparison to other structures of similar size at a related resolution range. We attribute this to poor order in parts of the monomers C and D. The model contains no Ramachandran outliers as identified by PROCHECK [36]. The overall G-factors which were used as a measure of the stereochemical quality of the model are 0.0 (PROCHECK) which is better than expected for the reported resolution. Many amino acid side chains, particularly in monomers A and B were modelled with alternative conformations. The residues Pro157, Pro306 and Ser329 are in the *cis* conformation in CelDZ1.

#### Overall structure and comparison to homologous enzymes

**Fig 5:**
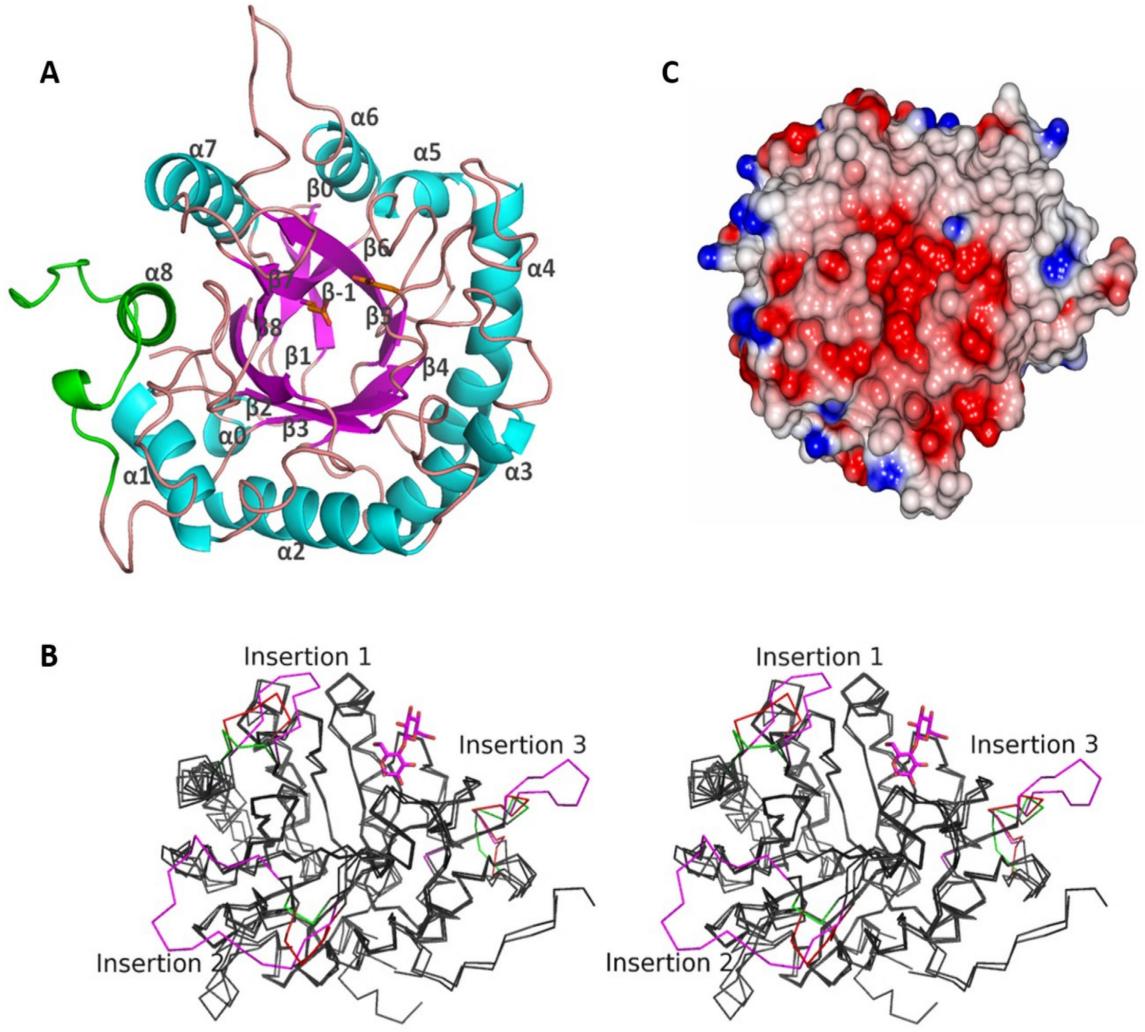
The structure of CelDZ1. **(A)** Folding of the CelDZ1α monomer is presented as a cartoon diagram and viewed from the solvent region towards the active site groove formed by the C-terminal ends of the β-strands of the (β/α)_8_-barrel. The α-helices, β-strands and loops are coloured in turquoise, magenta and pink, respectively. The carbohydrate-binding module (Fig 1), which contains helix α8 at the C terminus, is highlighted in green. The two catalytic residues are shown as stick models and secondary structural elements are labelled. The Met50 indicates the position of the first N-terminal residue defined in the electron density. The image was prepared using PyMol [40]. **(B)** A stereo representation of the superimposition of the monomers of CelDZ1, CelK and Cel5a displayed as grey carbon traces. The three different insertion regions are highlighted in red for CelDZ1, magenta for CelK and green for Cel5a. The cellobiose ligand bound to CelK is shown as a magenta stick model. The image was prepared using PyMol [40]. **(C)** The electrostatic potential surface of the CelDZ1 enzyme around the active site groove as viewed from the solvent region. The positive charge is shown in blue and the negative charge is shown in red. The extended active site groove, which crosses the monomer from left to right, is negatively charged disfavoring the binding of halogen ions thereby increasing halotolerance. The image was prepared with ccp4mg [41].

Although the asymmetric unit of CelDZ1 crystal contains four monomers, these do not form oligomers in the crystal according to PISA [38]. This is consistent with the apparent monomeric size of the protein that was observed by size exclusion chromatography (data not shown). CelDZ1 has an (β/α)_8_-barrel structure with two additional β-hairpins, one at its N terminus and another preceding helix α6 (Fig 5A). The C-terminal helix α8 is involved in the carbohydrate-binding motif. Its fold is similar to structures of other members of the subfamily 5–2 endoglucanases, which include the catalytic domain of the *Bacillus* sp. KSM-635 alkaline cellulase K (CelK; PDB code 1G0C; 58% amino acid sequence identity) [29] and the Cel5A cellulase from *Bacillus agaradherans* (PDB code 1H5V; 44% identity) [39]. Cel5A is a soluble protein with a somewhat shorter carbohydrate-binding motif at the C terminus. Several structures of Cel5A have been reported with bound ligands and inhibitors in order to structurally probe its catalytic mechanism [39]. CelK is a single domain from a multi-domain protein that is adapted to catalysis at alkaline pH, for which a structure with a bound ligand (cellobiose) has been reported [29].

Cel5A, CelK, and CelDZ1 proteins differ since the first is a soluble enzyme which is truncated at the N and C termini, CelK is part of a multi-domain protein and CelDZ1α is membrane-anchored in its native state. There are three regions where these enzymes differ from each other structurally (Fig 5B). A short connection between β4 and α4 in Cel5A is replaced by a longer loop in CelDZ1, which is even longer in CelK. At the beginning of helix α6, CelDZ1 has a small insertion relative to Cel5A, which forms a β-hairpin pointing towards the solvent, while CelK has a more extended loop at this position, which covers helices α5 and α6 from the solvent. After sheet β8, the linker going into the carbohydrate-binding motif is more extended in CelK and CelDZ1α in relation to the more compact Cel5A.

All three *cis*-peptide bonds in CelDZ1 are conserved in CelK and only one of these Trp262-Ser263 (equivalent to Trp328-Ser329 in CelDZ1) is conserved in Cel5A. This Trp residue forms an H-bond to the sugar substrate at the subsite -2. Interestingly, *cis*-Pro306 lies in the loop formed by residues 298–306, the equivalent of which undergoes significant induced fit motion upon sugar ligand binding in the subsites -1 and 1 in Cel5A [39]. Similarly to CelK [29], this loop adopts the active conformation in the absence of the sugar ligand, with the *cis*-Pro maintaining this structure.

All of the catalytic residues are conserved with Glu192 and Glu294 found on the C termini of the barrel strands β4 and β7, which are the acid/base and the nucleophile for catalysis, respectively. The cellobiose ligand used to define the sugar substrate-binding site is expected to bind in the -2 and -3 subsites of the enzyme in a similar way to that observed in the CelK and Cel5A enzyme structures. The amino acid residue Trp91 provides a stacking interaction with the glucose unit in the -3 subsite. Similarly, the conserved residue Trp237 is expected to form a stacking pair with the glucose unit at the 1 subsite. The conserved residues Trp328, Lys333 Glu335, His87, Tyr118 and Glu121 are all expected to provide hydrogen bond contacts to the glucose units of the cellulose molecule in the same manner as shown for the CelK and Cel5Α enzymes.

Whilst both the CelK and Cel5A enzymes have the distinct 2 subsite, where the residues Gln180 and His206 (Cel5A numbering) form H-bonds to the oxygens of the glucose unit at this position, neither of these residues are conserved in CelDZ1 where Thr239/Ala265 replaces the equivalent Gln/His residues. The two residues of CelDZ1 are unable to form a sugar-binding subsite and there are no apparent nearby residues capable of binding a sugar unit. CelDZ1 thus appears to be the first cellulose structure lacking the sugar-binding 2 subsite. Interestingly, only the closely related, uncharacterized glycosyl hydrolase family 5 from *Thermoanaerobacterium aotearoense* which has 95% sequence identity to CelDZ1, also appears to be lacking the Gln/His pair of residues, as determined by sequence analysis. All other homologues of CelDZ1 within the NCBI reference sequence database, including the next nearest uncharacterized homologous cellulase from *Caldanaerobacter subterraneus* MB4 (78% identity), contain the Gln/His residue pair which form the 2 subsite.

The CelK enzyme has been evolutionarily adapted to be stable in alkaline conditions. However, despite CelDZ1 having high sequence similarity to CelK, it is inactive above pH 8.0. Comparison of the overall amino acid composition between the two enzymes revealed a significant increase in positively charged arginine and lysine residues in CelDZ1 compared to CelK (39 compared to 24, respectively) resulting in higher predicted pI of CelDZ1 (5.7), when compared to that measured for CelK (4.5). Many of these positively charged residues are on the surface of CelDZ1. However, seven of the Arg-Asp ion pairs reported to be important for the alkaline adaptation of CelK [29] are reduced to five in CelDZ1. One of the residues responsible for the alkaline pH adaptation in CelK would appear to be His333, which is located at the position of Leu155 in CelDZ1 (also Leu in Cel5A). A deprotonation of this residue would make it unfavorable for a glucose unit to bind at the -1 subsite at physiological pH and below.

#### Structural features responsible for halotolerance

While halostability is quite a common feature of many thermostable enzymes, halotolerance appears to be less widespread. Halotolerance should be an important feature for maintaining activity of CelDZ1 which is predicted to be located on the outside of the cell membrane. It should be achieved by lowering the affinity of chloride and potassium/sodium ions to the enzyme active site, preventing their competition for substrate-binding sites. The calculation of the surface potential of CelDZ1 (Fig 5C) clearly demonstrates an overall negative charge in the active site channel which does not favor binding of chloride ions. A feature of other halophilic proteins is the presence of acidic amino acids on the surface of the protein [42, 43]. Monovalent cation binding sites are usually formed by a carboxylic site chain and at least one protein main chain carbonyl. Inspection of the ligand groove of CelDZ1 revealed no carbonyls exposed to solvent in the vicinity of carboxyl side chains which would form an alkaline ion-binding site. Although there is clearly a differential effect of Na^+^/K^+^ on thermo/halostability (Fig 4B), this lack of solvent-exposed carbonyl groups provides a possible explanation of the resistance of CelDZ1 to high concentrations of monovalent cations.

## Conclusion

In this study, a new thermotolerant and exceptionally halostable GH5 cellulase from an Icelandic *Thermoanaerobacterium* hot spring isolate was identified and characterized. This new enzyme, CelDZ1, is active at acidic pH, remains catalytically active at a wide temperature range for extended periods of time and exhibits biochemical characteristics that render it an attractive candidate as an additive to ‘enzyme cocktails’ that can be used for second-step processing of biomass or in other industrial processes that require robust enzymes that can withstand near-saturation salt concentrations combined with high temperatures. From a structural biology point of view, CelDZ1 is quite unique among its analogues in that it lacks the sugar-binding 2 subsite which is present in all known related enzymes.

## Materials and Methods

### Reagents and chemicals

All chemical reagents used in this study were purchased from Sigma-Aldrich unless stated otherwise. All molecular biology related products (restriction enzymes, protein markers, etc.) were from New England BioLabs.

### Enrichment culture

After the appropriate permission was issued by the National Energy Authority of Iceland, the outflow of a hot spring in Grensdalur, Iceland (64°01'53.4"N, 21°11'50.4"W) was collected together with the organic material surrounding the hot spring. The temperature of the water at the sampling site was around 40°C and the pH around 7. The sample was enriched anaerobically at 55°C, pH 7 with 0.01% yeast extract and 0.5% xylan as a carbon source. After several dilutions of the sample in xylan-containing medium, only rod-shaped microorganisms were visible under the microscope.

### Bioinformatic analysis

Genomic DNA was isolated from the aforementioned polysaccharide-enrichment culture and was submitted for deep sequencing using the Illumina platform (BGI, China) with a paired-end sequencing protocol providing >6 million reads of 90:90 base pairs in length. The raw sequencing reads were uploaded to our customized data analysis platform termed ANASTASIA (Automated Nucleotide Aminoacid Sequences Translational plAtform for Systemic Interpretation and Analysis) a metagenomics-analysis web platform dedicated to novel enzyme discovery through implementation of versatile, data-processing tasks (manuscript in preparation). Each of the following analyses exploited bioinformatic tools integrated as modular components in automated workflows encased into ANASTASIA. Assembly into contigs was performed using Velvet [44] (optimal k-mer value selected = 51, n50 = 184287). For the *de novo* prediction of coding sequences in the generated contigs, three different types of software were utilized, each based on a different machine-learning model: MetaGeneAnnotator [45], MetaGeneMark [46] and Prodigal [47]. The combined results of all three analyses consisted of about 3000 putative gene sequences, which were subsequently submitted to a homology analysis using BLASTp [48] against sequences deposited in both NCBI-nr and UniProt/Swiss-Prot [49]. The generated results were imported into a local MySQL database [50] connected with ANASTASIA through dedicated data-entry Python scripts, integrated in its environment, and comparative tables were created using appropriate search queries. These tables comprised the highest similarity scoring results from both databases for every single sequence including the corresponding EC numbers from the Uniprot/Swiss-Prot database. The sequences were also examined for Pfam domains using HMMER (hmmscan) against the Pfam-A database. The generated results from the HMMER analysis were also imported in the aforementioned MySQL database through other in-house Python scripts and were further queried in order to return sequences with domains related to cellulase activity. From the BLAST hits, the ones with the highest scoring homology to sequences with cellulase activity (EC number: 3.2.1.4) were selected from the UniProt/Swiss-Prot database and were compared with the corresponding hits from the NCBI-nr database. The sequence subsequently nominated as CelDZ1α was one of the hits considered of high interest as it showed 59% identity (e-value<0.001, query coverage 92%, positive percentage 73%) to an endo-1,4-beta-glucanase from *Bacillus akibai* (JCM 9157) in UniProt (Accession number: P06564.1). The corresponding hit in NCBI-nr had a 95% identity (e-value<0.001, query coverage 99%, positive percentage 97%) to a sequence annotated as glycosyl hydrolase family 5 from *Thermoanaerobacterium aotearoense* (Accession number: WP_014757289.1). It also showed two significant Pfam-A matches from the list of cellulase-related domains: (i) cellulase (glycosyl hydrolase family 5—ID: PF00150.13) and (ii) carbohydrate-binding domain (family 17/28—ID: PF03424.9). Further curation of the sequence included putative EC assignment by exploiting machine-learning based methodologies, namely EFICAz2.5 [51] and rpsBLAST against the PRIAM database [52]. Both software packages predicted an EC number of 3.2.1.4, which is in agreement with the UniProt/Swiss-Prot results.

### Plasmid construction

pET-CelDZ1α was constructed by amplifying *celDZ1a* from genomic DNA isolated from the enrichment culture by PCR using the forward primer 5’- AAAAATCTAGAAGGAGGAAACGATGAATAAATGGCATATTAACAAATGGTACTTTTTTGTAGG-3’ containing an *Xba*I site (underlined) and the reverse primer 5’AAAAACTCGAGTTAGTGGTGGTGGTGGTGGTGGTGGTGTTTTCCCATCGTCTCGCGAGAAATAGGTTTATAAGGAATTCCC-3’ containing an *Xho*I site (underlined) and a hexahistidine tag (doubly underlined), digested with XbaI and XhoI and inserted into similarly digested pET-28a(+) (Novagen). pET-CelDZ1 was constructed by replacing amino acids 2–27 of CelDZ1α with a hexahistidine tag. For this, *celDZ1* was amplified from pET-CelDZ1α using the forward primer 5’- AAAAATCTAGAAGGAGGAAACGATGCACCACCACCACCACCACAAAGATACATCTTTAACCTTTAGTAGTTATGATCGGG -3’ (*Xba*I restriction site underlined and the hexahistidine tag doubly underlined) and the reverse primer 5’-primerAAAAACTCGAGTTATTTTCCCATCGTCTCGCGAGAAATAGGTTTATAAGGAATTCCC-3’ containing an XhoI site (underlined). The correct sequence for all constructs was verified by standard DNA sequencing.

### Protein expression and purification

*E*. *coli* BL21(DE3) cells carrying the plasmid pET-CelDZ1 were grown in LB broth containing 50 μg/ml kanamycin at 37°C under constant shaking until the culture reached an optical density at 600 nm of about 0.5. At that point, the expression of *celDZ1* was induced by the addition of 0.2 mM isopropyl thio-β-D-galactoside (IPTG) followed by overnight incubation at 25°C with shaking. For CelDZ1 purification, the cells from a 500 mL culture grown in a 2 L shake flask were harvested, washed, re-suspended in 10 mL equilibration buffer NPI10, and lysed by brief sonication steps on ice. The cell extract was clarified by centrifugation at 10,000×g for 15 min at 4°C and the supernatant was combined with 0.5 mL Ni-NTA agarose beads (Qiagen) and shaken mildly for 2 h at 4°C. The mixture was then loaded onto a 5 mL polypropylene column (Thermo Scientific), the flow-through was discarded, and the column was washed with double the whole column volume of NPI20 wash buffer. CelDZ1 was eluted using NPI200 elution buffer. All buffers used for purification were prepared according to the manufacturer’s protocol. Imidazole was subsequently removed from this protein preparation using a Sephadex G-25 M PD10 column (GE Healthcare). Protein concentration was estimated according to the assay described by Bradford [53] using bovine serum albumin as a standard. The purified protein was visualized by SDS-PAGE analysis and Western blotting.

### Enzyme activity assays

For the detection of cellulolytic activity by zymography, 12% SDS–PAGE gels were enriched with 0.25% carboxymethyl-cellulose (CMC). All procedures and materials used were standard, except that the samples were not boiled prior to gel loading. After electrophoresis, the gel was gently shaken for 30 min in 50 mM Tris buffer pH 7 with 2% Triton X-100, then for 30 min in 50 mM Tris buffer pH 7, then for 3 h in 50 mM potassium phosphate pH 7 at 70°C, and finally stained with a 1% Congo red solution in water for 40 min. Destaining was carried out with 1 M Tris buffer pH 7 for 15 min at room temperature, followed by setting the dye in 1 M MgCl_2_ [18, 54].

For the biochemical characterization of CelDZ1, the cellulolytic activity of the enzyme was determined by quantification of the amount of reducing sugar released from the substrate using the 3,5–dinitrosalicylic acid (DNS) method [27]. One unit (U) of activity was defined as the quantity of enzyme required to release 1 μmol of reducing sugar per min. The standard reaction consisted of 50 mM phosphate buffer at pH 5 and 1% (w/v) CMC as the substrate, and 3 μg/mL enzyme. Enzyme reactions were carried out on a MJ Research thermal cycler at 70°C for 5 min unless stated otherwise. The reactions were terminated by the addition of equal volume of DNS and the mixture was boiled for 5 min to develop the colour occurring due to the reaction with reducing sugars. Enzymic activity was recorded by measuring the absorbance at 540 nm. For the determination of the enzyme’s optimal pH, the reactions were carried out at 40°C in 50 mM acetate, phosphate, Tris-HCl and glycine buffers for pH values 4–6, 7, 8–9 and 10, respectively. The temperature profiling of CelDZ1 was performed by incubating the standard reaction at temperatures ranging from 40 to 90°C. Kinetic parameters were determined by using the standard reaction format with CMC concentrations ranging from 0.3 to 3%. Data analysis and curve fitting was performed using the Graphpad Prism 5 software. For the substrate specificity experiments, CMC was replaced in the standard reaction by other soluble polysaccharides. For the insoluble substrates such as Avicel and filter paper the reaction time was 24 h, and the enzyme concentration was increased 10 fold. In the thermostability studies, CelDZ1 was replaced in the standard assay by the pre-incubated enzyme in various temperatures and for different time intervals. Halostability and halotolerance studies were also executed in the standard reaction with the only difference being the addition of salts. The same applies for the metals and denaturing agents studies. All measurements were obtained from at least three independent experiments carried out in triplicates.

Thermal denaturation analysis by differential scanning fluorimetry was conducted using a 10X SYPRO Orange (Thermo Scientific) concentration mixed with the enzyme at 10 μg/mL in 50 mM sodium acetate buffer, pH 5. The samples were incubated at a temperature range of 30–100°C on a Biorad IQ5 real time PCR machine in triplicate. The fluorescence intensity was monitored by increasing the temperature in 1°C increments, with a pause time of 1 min, from 30 to 100°C. The melting point (T_m_) of the enzyme was identified from the midpoint of the melting curve. The data were analyzed as presented by the Biorad iQ5 Optical System Software.

### Crystallization

Prior to protein crystallization, CelDZ1α was further purified using a calibrated Superdex 200 HiLoad 16/60 gel filtration (GF) column (GE Healthcare) and was eluted with 1 column volume in a buffer of 25 mM Tris-HCl, 0.1 M NaCl, pH 7.5 at 1.0 ml/min. The isolated enzyme was concentrated to ~15 mg/ml using a 10 kDa Vivaspin membrane (Vivaproducts) and microbatch crystallization trials were set up using an Oryx 6 crystallization robot (Douglas Instruments) using the The Stura Footprint Screen™ + MacroSol™ HT-96 screen (Molecular Dimensions). The droplet contained a 50:50 ratio of protein solution to screen and was covered with Al’s oil (50:50 mix of silicon and paraffin oils) before being stored at 20°C and was regularly checked for growth of crystals using a light microscope. Crystals appeared within one week, grown from 50 mM sodium HEPES pH 7.5, 10% v/v, 100 mM magnesium chloride hexahydrate and 10% v/v 2-propanol. Crystals were cryo-cooled in a solution containing 35% PEG400, 30% of the gel filtration buffer solution and 35% of the crystallization condition.

#### X-ray data collection and structure solution

Data were collected on beamline I04-1 at the Diamond Synchrotron light source (Didcot, UK) at 100 K in a stream of gaseous nitrogen using a Pilatus detector (Dectris). Data were processed and scaled using XDS [55] and AIMLESS [56] in the Xia2 [57] pipeline. All further data and model manipulation was carried out using the CCP4 suite of programs [58]. Phases for the native structure were determined using the molecular replacement method (MR) implemented in MOLREP [59] using the monomer of CelK as a model [29]. The rotation function was calculated with an integration radius of 36 Å at a resolution of 2.2 Å and gave four prominent rotation peaks of 16, 14, 8 and 7 σ height. The translation search has allowed the positioning of four monomers of CelDZ1 in the asymmetric unit. The MR solution was rebuilt using the ARP/wARP automated refinement procedure [60]. This was followed by manual model building in Coot [61] and refinement using Refmac5 [62]. To build the poorly defined monomers C and D the non-crystallographic averaging implemented in DM [63] was used. The phases from density modification were used as input into REFMAC5 phased refinement [64].
